# Hyperspectral imaging and indocyanine green fluorescence angiography in acute mesenteric ischemia: A case report on how to visualize intestinal perfusion

**DOI:** 10.1016/j.ijscr.2021.105853

**Published:** 2021-04-01

**Authors:** Matthias Mehdorn, Sebastian Ebel, Hannes Köhler, Ines Gockel, Boris Jansen-Winkeln

**Affiliations:** a Department of Visceral, Transplant, Thoracic and Vascular Surgery, University Hospital of Leipzig, Leipzig, Germany; b Department of Diagnostic and Interventional Radiology, University Hospital of Leipzig, Leipzig, Germany; c Innovation Center Computer Assisted Surgery (ICCAS), University of Leipzig, Leipzig, Germany

**Keywords:** Acute mesenteric ischemia, Intraoperative imaging, Image-guided surgery, Hyperspectral imaging, Indocyanine green fluorescence angiography, Case report

## Abstract

**Introduction and importance:**

Acute mesenteric ischemia is a challenging acute condition which is often caused by occlusion of an intestinal vessel. Therapeutic algorithms include revascularization of the occluded vessel and a surgical procedure to remove necrotic intestine. Sometimes necrotic intestine is hard to identify visually. Therefore, tools such as hyperspectral imaging (HSI) and indocyanine green fluorescence angiography (ICGFA) might be helpful for objective intraoperative evaluation of intestinal perfusion.

**Case presentation:**

We present a case of an 80-year-old woman with an acute superior mesenteric artery occlusion and subsequent intestinal gangrene. After endovascular arterial revascularization, we performed an explorative laparotomy in which we assessed intestinal perfusion by HSI and ICGFA. Both HSI and ICGFA showed a sharp perfusion borderline in the proximal jejunum. The distal intestine showed low tissue oxygenation (HSI) and inhomogeneous perfusion (ICGFA).

**Clinical discussion:**

Both methods showed reproducible results for tissue perfusion and, thus, could provide additional information on the extent of necrotic bowel with need for resection. Therefore, both modalities might be used in future image-guided surgery in cases of acute mesenteric ischemia where visual discrimination of intestinal perfusion is challenging in order to resect as much bowel as necessary to improve patient outcome. Both methods exert different strengths: i.e. ICGFA is real-time angiography, whereas HSI may expose intestinal necrosis in spectroscopy.

**Conclusion:**

We show, for the first time, simultaneous imaging of HSI and ICGFA in a case of acute mesenteric ischemia. Both imaging modalities reveal similar results reliably concerning intestinal perfusion.

## Highlights

• First case of HSI and ICG in Acute mesenteric ischemia.
• Similar results of moth modalities with regard to well perfused intestine.
• ICG shows exact vascular blood supply and intestinal perfusion.
• Hyperspectral imaging can distinguish necrotic and vital intestinal segments in AMI.
• Combined use helps surgeons to evaluate intestinal perfusion intraoperatively in AMI.

## 1 Introduction

Acute mesenteric ischemia (AMI) is a life threatening and challenging acute condition with high mortality of up to 50% and a considerable morbidity, such as short bowel syndrome. Mostly, it is caused by superior mesenteric artery (SMA) occlusion [1]. Open surgical or endovascular revascularization are both therapeutic options, depending on the patient’s condition [2]. After a potential revascularization procedure, an explorative laparoscopy or laparotomy is performed to assess the intestine for bowel gangrene that needs to be resected in a damage control style [3,4]. For the surgeon, the most challenging part is to discriminate vital and non-vital intestine to ensure the best outcome [5], as necrotic intestinal segments sometimes do not appear necrotic in the first place. Therefore, objective tools are needed during surgical exploration to assess intestinal perfusion and viability.

There are two possible tools available to asses intestinal perfusion during surgery for AMI nowadays: Hyperspectral imaging (HSI) [6] and Indocyanine green fluorescence angiography (ICGFA) [5,7,8]. Today, large-scale data are lacking on the specific advantages of each modality as no publications exist comparing both methods in AMI. We have previously reported the simultaneous use in elective colorectal surgery [9] with similar results of visualizing intestinal perfusion.

We report a case from our tertiary referral hospital of an 80-year-old woman with acute mesenteric ischemia due to SMA occlusion in who we were able to record both imaging techniques during the primary explorative laparotomy.

The case is reported in accordance with the SCARE 2020 guidelines of reporting surgical cases [10].

## 2 Presentation of case

**Fig. 1:**
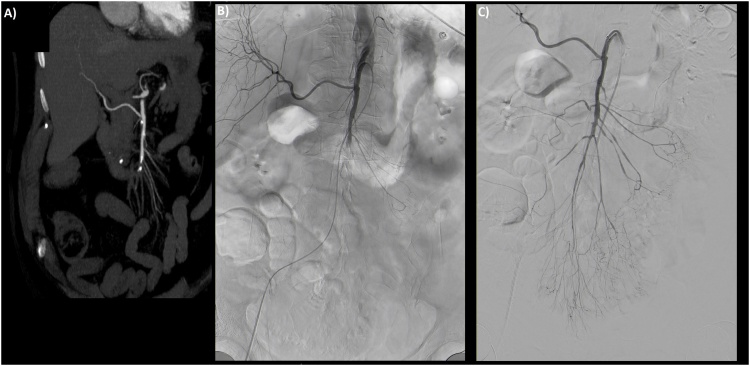
Radiologic imaging of the superior mesenteric artery (SMA) occlusion and subsequent endovascular thrombectomy. a) Contrast enhanced CT angiography with distal SMA occlusion, b) Angiography of the SMA before thrombectomy with perfusion of the proximal jejunal branches and an aberrant origin of the right hepatic artery. c) Successful recanalization of the SMA and its branches.

**Fig. 2:**
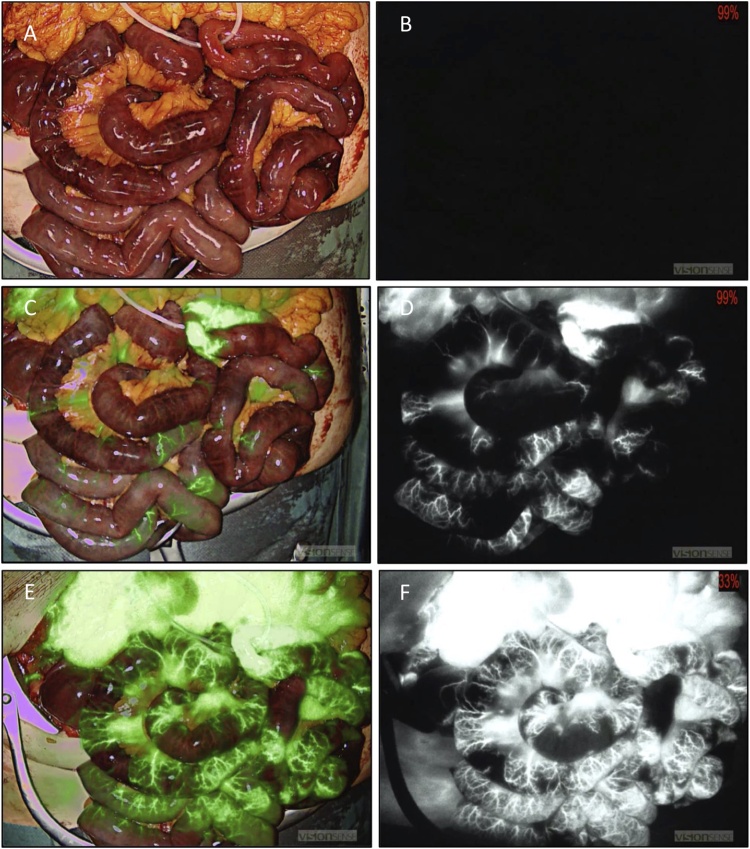
ICG angiography of the small bowel with a/b before injection of ICG, c/d 30 s after and e/f 60 s after ICG injection. Left column represents the green overlay images, right column the infrared grayscale images. The red number in the upper right corner of the infrared pictures represents the necessary excitation required to detect the maximum fluorescence signal (the fluorescence intensity dramatically increases from 30 to 60 s). At the top center of the images the vital jejunal loop, marked with a vessel loop, displays rapid ICG fluorescence. The rest of the small intestine does not present fluorescence of the intestinal wall.

**Fig. 3:**
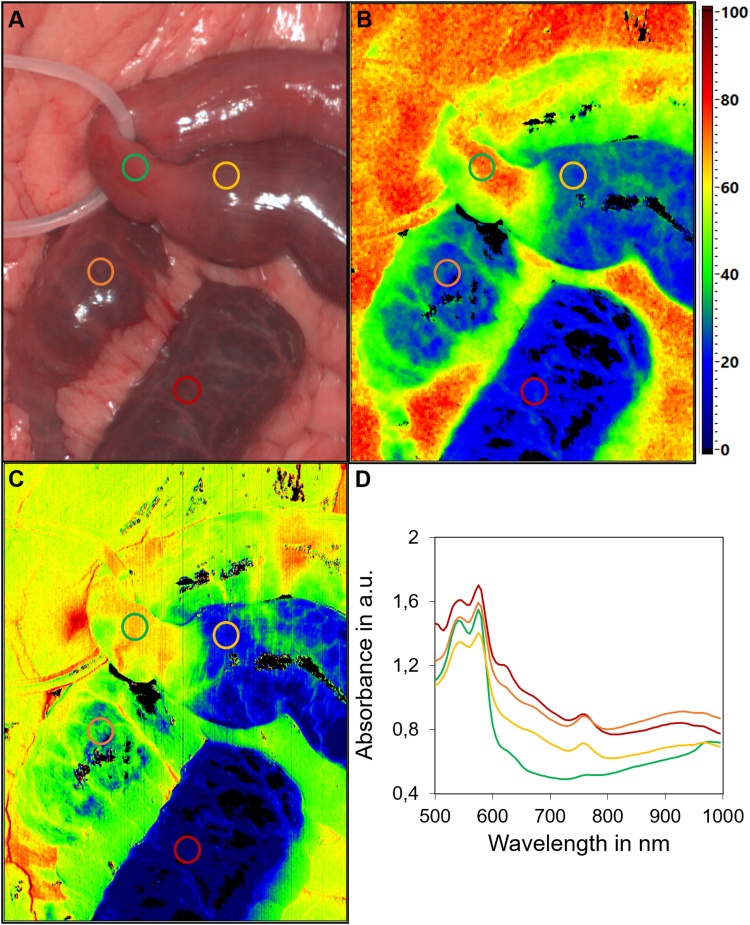
HSI of the macroscopic perfusion borderline. The jejunal loop is marked with a vessel loop. In the false-color images, the respective quantitative value can be deduced from the scale at the right of the picture. a) color image; b) Tissue oxygenation (StO_2_).; c) Near infrared perfusion index (NIR-PI); d) Absorbance spectra of the colored regions of interest in the picture. The spectra show a peak at 630 nm, representing necrotic tissues, for all regions but the one proximal to the perfusion border line.

An 80-year-old woman with a known cardiovascular medical history who was living on her own, presented to our emergency room via ambulance with upper abdominal pain and vomiting for the last 12 h. As the troponin T was massively elevated, primary clinical suspicion was an untypical presentation of myocardial infarction. The subsequently performed coronary angiography could not identify any coronary pathologies. Because of the persistent abdominal pain, the blood lactate of 15 mmol/l and 23 exp9/l leucocytes, the surgeon on call was involved. Clinical examination showed diffuse abdominal pain with no localized tenderness. The abdominal CT angiography demonstrated a distal occlusion of the SMA with no radiographic signs of advanced bowel necrosis (Fig. 1a). In accordance with our institutional standard operating procedures, the patient was taken for endovascular revascularization after exclusion of radiographic signs of bowel necrosis. The occlusion of the SMA was successfully reopened using thrombus aspiration and local lysis (Fig. 1b and c). After revascularization, we (MM and BJW) performed an explorative laparotomy in which we found a vital proximal jejunum, about 50 cm of potentially necrotic jejunum and a gangrenous distal jejunum, ileum and right colon (Fig. 2a). The visible border line between the vital and questionably necrotic intestine was localized with a vessel loop. ICGFA and HSI were performed in the way described before [9]. The ICGFA revealed a rapidly increasing fluorescence signal in the proximal jejunum, including the vessels and the intestinal tissue. In the distal intestinal segments only segmental fluorescence of the supplying vessels could be detected, with no fluorescence of most of the intestinal tissue (Fig. 2c–f). We performed HSI of the questionable perfusion border, which showed an exact and very defined edge of perfusion at the location of the vessel loop with tissue saturation of more than 80% (Fig. 3b) and a near-infrared perfusion index (NIR-PI) of 70% (Fig. 3c). The questionably necrotic segment showed very low tissue saturation and NIR-PI of about 10–20% (Fig. 3b, c). The postoperative analysis of the emission spectra of those segments demonstrated peaks at about 630 nm as an indication for necrotic tissues [6], beginning directly after the detectable perfusion border (Fig. 3d). Interestingly, the bowel’s mesentery was nearly unaffected by the ischemia, showing high tissue saturation and NIR-PI. Hence, the macroscopic borderline between vital and questionably necrotic bowel was confirmed by HSI and ICGFA.

As data about HSI and ICG in mesenteric ischemia were lacking at that time and with a certain hope that the questionably necrotic intestine would recover after revascularization, we decided to refrain from resection of the macroscopically less perfused segment and to judge it in a second look surgery 48 h later. During the early post-operative course, the patient’s condition deteriorated, so that we decided to perform an early second-look almost 20 h after the initial procedure. We found bowel necrosis of the previously less perfused segment and went on with resection. After stabilization of the initial multi-organ failure, a third look could be performed in which the remaining intestine was vital and an anastomotic stoma was performed [11]. During the later stages of the in-hospital recovery period the patient suffered from acute renal failure because of her high-output stoma. After a total of 35 days she was transferred to a rehabilitation facility. During the following months, the leading problem remained the short bowel syndrome with high-output stoma, resulting in several readmissions, parenteral nutrition support and teduglutide application and almost 6 months after the initial event, the ostomy was reversed. In the end, the patient still suffers from short bowel syndrome with diarrhea-like defecation, but she still is able to live on her own.

## 3 Discussion

In this case, we demonstrate our first experience of simultaneous application of HSI and ICGFA to evaluate the intestinal perfusion and viability in a case of AMI due to SMA occlusion.

Indocyanine green (ICG) is a fluorescence dye which is activated by the specific wavelength of 805 nm and its emission spectra are detected with the respective device (i.e. VisionSense3 system, Medtronic, Meerbusch, Germany). To perform real-time angiography, the dye is injected intravenously and within a minute fluorescence signals occur, depending on tissue vascularization and circulation time. We have described the technique in detail before [9]. Several different visualizations are displayed in video rate by the ICGFA system (red-green-blue; infrared; grayscale; green overlay) and thus tissue perfusion can be evaluated in a real-time fashion. It has been shown that using ICGFA can help to reduce anastomotic leakage in colorectal surgeries [12,13]. The use of ICGA in AMI has rarely been reported but it seems that ICG might be suitable to visualize intestinal perfusion differences in non-occlusive mesenteric ischemia [8] as well as after successful revascularization [5]. The only cohort study of 52 patients with AMI in which the use of ICG has been evaluated revealed a change in surgical strategy in 6 (11%) of the patients with two false-negative angiographies [7].

For HSI we use the TIVITA tissue suite (Diaspective Vision GmbH, Am Salzhaff, Germany). The exact technical details of this imaging method have been described previously [9]. Briefly, the HSI system displays several false-color images representing physiological tissue parameters, such as oxygenation (StO_2_, Fig. 3b) or NIR PI (Fig. 3c) and a reconstructed color image. Imaging takes a few seconds, is contactless and does not require any drugs. After primary imaging and analysis, detailed spectroscopy of several regions of interest can be performed and the absorbance spectra are displayed (Fig. 3d). A specific peak at 630 nm can be found in necrotic tissues. In comparison to ICGFA, even less evidence exists for the use of HSI in AMI. We have recently reported our experience with HSI in a case series of AMI [6]. We could discriminate intestinal tissues by their perfusion as the StO_2_ and NIR PI differed remarkably between visually well and poorly perfused intestinal segments. Additionally, the segments with impaired perfusion also showed spectral peaks at 630 nm, representing tissue necrosis. These peaks could be found directly distal of the perfusion limit (Fig. 3d), actually requiring surgical resection at the moment of primary surgery.

During the explorative laparotomy of our case, HSI and ICGFA were able to clearly delineate the perfusion border of the jejunum at the time of primary operation. In accordance with the preoperative angiography, the proximal jejunum was vital, followed by 50 cm of potentially necrotic bowel. This was represented by visibly perfused vessels in ICGFA and by high StO_2_ and NIR PI values in HSI. Similarly, both methods were able to show the missing microperfusion. Both measurements reflect the low ischemia tolerance of the intestinal tissues compared to the adipose tissues of the mesentery. Furthermore, ICGFA demonstrated a loss of fluorescence signal of the smaller intestinal vessels, possibly due to local thrombosis after SMA occlusion and subsequent cessation of the local blood supply. This might be the reason for the increased hemoglobin content in ischemic tissues, as reported previously by our group [6].

There are two main points to consider when applying HSI and ICGFA: On one hand, ICGFA heavily relies on the correct administration of the drug (i.e. injection dosage and velocity, circulation time). Otherwise it might result in false-negative imaging. On the other hand, the most recent version of the TIVITA® Tissue suite does not provide real-time analysis of tissue perfusion at video-rate.

The retrospective analysis of this case brings up several points: The initial clinical suspicion of myocardial infarction, brought up by high troponin levels, was delaying the correct diagnostic measures. But this reflects the every-day challenge of diagnosing acute mesenteric ischemia, as symptoms may vary. The elevated cardiac markers were probably due to a septic cardiomyopathy. Furthermore, the cardiologists could have performed an angiography of the SMA while performing the coronary angiography to exclude mesenteric ischemia. Unfortunately, it took some more hours until the revascularization of the SMA took place. Today, controversy exists on whether open or endovascular revascularization is preferable [4]. As we have very experienced interventional radiologists and angiologists, our preferred approach is endovascular. The intestinal perfusion border line was the same for macroscopic impression, HSI and ICGFA. As the remaining bowel would have been some 70 cm of jejunum, we decided to leave the questionably vital bowel of the jejunum in place. In the retrospective view, we should have performed the radical resection as this segment proved necrotic in the second look surgery. Our hope remains, that HSI or ICGFA may help to reveal perfusion borders in cases of AMI that are not as obvious as in this case.

Although data on intraoperative imaging in AMI are scarce, the results are encouraging with regard to improving patients’ outcome. Further large-scale and prospective studies are needed to gain more knowledge and to establish intraoperative imaging during surgery for AMI.

## 4 Conclusion

In our case, we show that HSI and ICGFA both display intestinal perfusion in AMI in a similar quality with HSI having the advantage of visualizing necrotic tissues through spectroscopy and ICG in performing real-time angiography. We provide evidence that a useful tool for the management of AMI might be available in the near future. Hence, further studies are needed to evaluate the usefulness of HSI and ICGFA in AMI to improve patients’ outcomes.

## Declaration of Competing Interest

Hannes Köhler was employee of Diaspective Vision. The other authors have nothing to declare.

## Sources of funding

The project did not receive any funding.

## Ethical approval

N/A.

## Consent

Written informed consent was obtained from the patient for publication of this case report and accompanying images. A copy of the written consent is available for review by the Editor-in-Chief of this journal on request.

## Author contribution

MM and JW performed the surgery, acquired data, analyzed data and drafted the manuscript.

SE performed the angiography, provided radiologic imaging and critically revised the manuscript.

HK performed HSI analysis and critically revised the manuscript.

IG analyzed data and critically revised the manuscript.

All authors have read and approved the final manuscript.

## Registration of research studies

Not applicable.

## Guarantor

Matthias Mehdorn, M.D.

## Provenance and peer review

Not commissioned, externally peer-reviewed.
